# Genomic Investigation of Methicillin-Resistant *Staphylococcus aureus* ST113 Strains Isolated from Tertiary Care Hospitals in Pakistan

**DOI:** 10.3390/antibiotics10091121

**Published:** 2021-09-17

**Authors:** Nimat Ullah, Hamza Arshad Dar, Kanwal Naz, Saadia Andleeb, Abdur Rahman, Muhammad Tariq Saeed, Fazal Hanan, Taeok Bae, Amjad Ali

**Affiliations:** 1 Atta-ur-Rahman School of Applied Biosciences (ASAB), National University of Sciences and Technology (NUST), Sector H-12, Islamabad 44000, Pakistan; nullah.phdabs12asab@asab.nust.edu.pk (N.U.); darhamza000@gmail.com (H.A.D.); kanwal.naz03@gmail.com (K.N.); saadia.andleeb@asab.nust.edu.pk (S.A.); a.rahman@asab.nust.edu.pk (A.R.); 2 Research Center for Modeling and Simulation (RCMS), National University of Sciences and Technology (NUST), Islamabad 44000, Pakistan; tariq@rcms.nust.edu.pk; 3 Department of Pathology, Saidu Medical College, Saidu Sharif, Swat 19200, Pakistan; drfhanan@gmail.com; 4 Department of Microbiology and Immunology, Indiana University School of Medicine-Northwest, 3400 Broadway, Gary, IN 46408, USA

**Keywords:** antibiotic-resistance, comparative genome analysis, methicillin-resistant *Staphylococcus aureus*, multi-locus sequence type, ST113, whole-genome sequencing

## Abstract

Methicillin-resistant *Staphylococcus aureus* (MRSA) is a multi-drug resistant and opportunistic pathogen. The emergence of new clones of MRSA in both healthcare settings and the community warrants serious attention and epidemiological surveillance. However, epidemiological data of MRSA isolates from Pakistan are limited. We performed a whole-genome-based comparative analysis of two (P10 and R46) MRSA strains isolated from two provinces of Pakistan to understand the genetic diversity, sequence type (ST), and distribution of virulence and antibiotic-resistance genes. The strains belong to ST113 and harbor the SCCmec type IV encoding *mecA* gene. Both the strains contain two plasmids, and three and two complete prophage sequences are present in P10 and R46, respectively. The specific antibiotic resistance determinants in P10 include two aminoglycoside-resistance genes, *aph(3’)-IIIa* and *aad(6)*, a streptothrin-resistance gene *sat-4*, a tetracycline-resistance gene *tet(K)*, a mupirocin-resistance gene *mupA*, a point mutation in *fusA* conferring resistance to fusidic acid, and in strain R46 a specific plasmid associated gene *ant(4’)-Ib*. The strains harbor many virulence factors common to MRSA. However, no Panton-Valentine leucocidin (*lukF-PV/lukS-PV*) or toxic shock syndrome toxin (*tsst*) genes were detected in any of the genomes. The phylogenetic relationship of P10 and R46 with other prevailing MRSA strains suggests that ST113 strains are closely related to ST8 strains and ST113 strains are a single-locus variant of ST8. These findings provide important information concerning the emerging MRSA clone ST113 in Pakistan and the sequenced strains can be used as reference strains for the comparative genomic analysis of other MRSA strains in Pakistan and ST113 strains globally.

## 1. Introduction

*Staphylococcus aureus* (*S. aureus*) is a Gram-positive opportunistic pathogen, causing a variety of pathological conditions, including skin and soft tissue infections, toxin-mediated syndromes, abscesses, necrotizing pneumonia, endocarditis, and bloodstream infection [1,2,3]. *S. aureus* has developed resistance against most of the routinely used antibiotics (e.g., beta-lactams, macrolides, and aminoglycosides) by acquiring antibiotic-resistant genes and less frequently by chromosomal gene mutations [4,5]. In the 1940s, a penicillin-resistant *S. aureus* was reported for the first time, and by the 1960s *S. aureus* also developed resistance against methicillin by acquiring the methicillin resistance genes (*mecA*) and gradually developed resistance against almost all beta-lactam antibiotics [6]. The pathogenicity and versatility of methicillin-resistant *S. aureus* (MRSA) to evade the host immune responses can be attributed to its ability to express multiple virulence factors, including enterotoxins, toxic shock syndrome toxin, nucleases, proteases, hemolysins, leucocidins, and fibronectin-binding proteins [7]. These virulence factors are mostly involved in adherence, colonization, and invasion abilities, allowing *S. aureus* to avoid host immune defense and to promote pathogenicity [8]. The presence of these virulent factors makes MRSA more virulent and difficult to treat [9].

The emergence of new antibiotic resistance in MRSA strains have been linked largely with hospital setting (HA-MRSA). However, recently, the dissemination of antibiotic resistance has also been reported in the community (CA-MRSA), which is often associated with the misuse of antibiotics [10,11]. This adaptation and evolution of MRSA is primarily associated with the mobile genetic element (MGE) called staphylococcal cassette chromosome *mec* (SCC*mec*). The SCC*mec* elements harbor methicillin-resistance gene (*mecA*), and among thirteen reported SCC*mec* types, SCC*mec* types IV and V are predominantly found in CA-MRSA, whereas SCC*mec* types I, II, and III are associated with HA-MRSA [12,13,14]. Among the genotypic methods, multi-locus sequence typing (MLST) has widely been used to determine the population structure and to examine the slow-evolving genomic core [15]. MLST coupled with SCC*mec* typing is also used to investigate the global spread of different clones of MRSA [16]. The knowledge of genome-level characteristics of the emerging strains, their resistance patterns, the spectrum of infections, and the identification of MRSA clones circulating in a geographical region may greatly help in finding the potential treatment options.

During the past decade, the prevalence of MRSA infections has increased from 35.9% to 66.7% in Pakistan [17]. The data of epidemiological typing of MRSA isolates from Pakistan are limited and very few studies have reported the distribution of MRSA clones. These studies reported that ST239 and ST8 clones are predominantly present among MRSA isolates in Pakistan [17,18,19], though ST1, ST217, ST1175, ST113, ST30, ST772, ST15, ST503, ST1413, and ST291 were also observed to be circulating in Pakistan [17,18,19,20,21]. However, these results are based on pulsed-field gel electrophoresis (PFGE), multi-locus sequence typing (MLST), and *spa* typing. None of these studies performed whole-genome sequencing of MRSA strains, which makes it difficult to compare MRSA strains in Pakistan to those in other countries.

In this study, we performed whole-genome sequencing of MRSA strains ST113 isolated from Pakistan and identified plasmid replicons, phage regions, and genes associated with antibiotic resistance and virulence. In addition, we determined the MLST and whole-genome single nucleotide polymorphisms (SNP)-based diversity of the sequenced strains with global MRSA strains (*n* = 252) and performed a genome-wide comparison of orthologous groups (COGs) with their phylogenetically related strains M51 ST1516 (CP030137.1), SVH7513 ST612 (CP029166.1), and USA300_2014.C02 ST8 (CP012120.2), since no draft or complete genome of ST113 strains is available in the public domain according to Pathosystems Resource Integration Center (PATRIC: https://www.patricbrc.org/, accessed on 29 March 2021). 

## 2. Results

### 2.1. Phenotypic Antibiotic Resistance of the Isolates

The strains were resistant to ampicillin, methicillin, oxacillin, gentamicin, streptomycin, erythromycin, clindamycin, meropenem, linezolid, and fusidic acid and were susceptible to chloramphenicol and vancomycin (Table 1). The strain P10 was also resistant to cefepime and R46 to rifampicin. The isolates produced a 500-bp amplified product of the *mec*A gene, which confirmed the MRSA nature and were whole-genome sequenced.

### 2.2. Genomic Characteristics

The de novo assembly of P10 and R46 Illumina reads generated 90 and 72 contigs (>500 bp), respectively, and a GC content of 32.7%. The N_50_, N_75_, and L_50_ values of the P10 genome are 84,395, 52,815, and 10, respectively, and the longest contig is 294,580 bp in length. Meanwhile, the N_50_, N_75_, and L_50_ values of the R46 genome are 84,399, 50,022, and 11, respectively, and the longest contig is 261,096 bp (Table 2). The genome size of P10 is 2,955,291 bp, and that of R46 is 2,822,631 bp. The number of predicted CDS is 3031 in P10 and 2827 in R46. P10 harbors 54 tRNA genes and nine rRNA genes, while R46 harbors 55 tRNA genes and 10 rRNA genes (Table 2). 

### 2.3. Genome-Based Typing and Mobile Genetic Elements

The genome analysis revealed that the strain P10 and R46 belong to ST113 and carry SCC*mec* type IV. The sequence analysis revealed that the P10 strain belongs to *spa*-type t064 and R46 has an unknown *spa*-type (Table 2). PlasmidFinder identified two plasmids in strain P10 having 99.78% and 99.72% similarity with *S. aureus* strain ST228 plasmid pI5S5 (HE579068.1) and *S. aureus* strain ER01881.3 plasmid unnamed1 (CP030577.1), respectively. The strain R46 contains one *S. aureus* plasmid pRM27 (KT780704.1) with 100% similarity and one *S. epidermidis* isolate BPH0662 plasmid (LT614820.1) with 99.83% similarity (Table 3). Three complete phages of length 42 kbp, 48.4 kbp, and 74.2 kbp were found in the strain P10 and two complete phages having genome size 55.6 kb and 71.4 kb in the strain R46 (Table 4).

### 2.4. Genes-Associated with Antibiotic Resistance and Virulence

**Figure 1:**
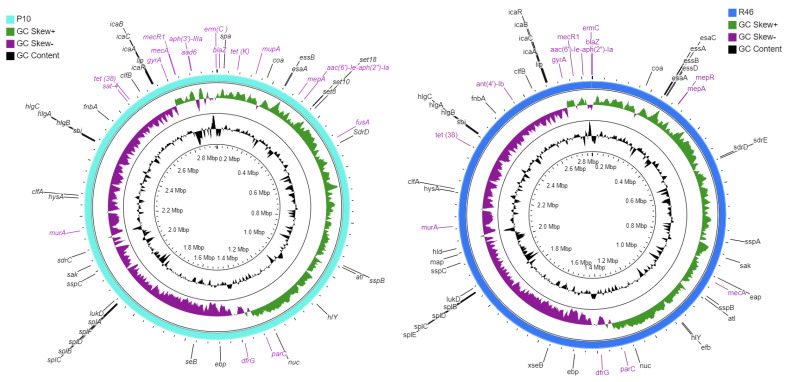
Circular visualization of P10 and R46 genomes via CGViewer, showing antibiotic resistance determinants (labeled in purple) and virulence genes (labeled in black).

The strains shared the following antibiotic resistance determinants: *blaZ*, conferring beta-lactam resistance by the enzymatic inactivation of antibiotics; the *mecA* gene conferring methicillin resistance by antibiotic target alteration, *mecR1*; an additional methicillin-resistance gene which interacts with beta-lactam antibiotics and upregulates *mecA*/*mec1* operon, aminoglycoside resistance gene *aac*(*6′*)*-Ie-aph*(*2″*)*-Ia,* conferring resistance by antibiotic enzymatic modification; glycylcycline resistance gene *mepA* and tetracycline resistance gene *tet(38)* conferring resistance via transport of antibiotics out of the cell, with a point mutation conferring antibiotic-resistance genes; *gyrA* and *parC* conferring resistance to fluoroquinolones via antibiotics target alteration; the trimethoprim resistance gene *dfrG* and the *murA* gene conferring resistance to fosfomycin; and a plasmid-associated resistance gene *erm(C)*, which causes antibiotic target replacement in the presence of drugs including lincosamide, macrolide, and streptogramin. Two aminoglycoside-resistance genes, *aph(3’)-IIIa* and *aad(6)*, and a streptothrin-resistance gene, *sat-4,* confer resistance by the inactivation of antibiotics; antibiotic efflux gene *tet(K)* conferring tetracycline-resistance; *mupA,* conferring mupirocin-resistance by antibiotic target alteration; and a point mutation in *fusA* conferring resistance to fusidic acid via antibiotic target alteration were present in strain P10. On the other hand, a plasmid-associated gene, *ant(4’)-Ib*, which causes resistance to aminoglycosides by inactivation of antibiotics, was found in strain R46 (Table 1 and Figure 1).

The virulence genes analysis, however, predicted many common genes in the studied strains. These include autolysin (*atl*), fibrinogen, and fibronectin-binding protein (*efb* and *fnbA*), cell wall-associated fibronectin-binding protein (*ebh*), Ser-Asp rich fibrinogen-binding proteins (*sdrC,* and *sdrD*), staphylococcal protein A (*spa*), cysteine protease (*sspB* and *sspC*), serine V8 protease (*sspA*), staphylocoagulase (*coa*), thermonuclease (*nuc*), type VII secretion system (*esaA*, *esaB*, *esaD*, *esaE*, and *esaG*), alpha-hemolysin (*hly*/*hla*), delta-hemolysin (*hld*), gamma-hemolysin (*hlgA*, *hlgB*, and *hlgC*), Enterotoxin B (*seb*), and Leukocidin D (*lukD*) genes (Figure 1). The strain R46 has an additional Ser-Asp rich fibrinogen-binding protein (*sdrE*). However, no Panton-Valentine leucocidin (*lukF-PV*/*lukS-PV*) or toxic shock syndrome toxin (*tsst*) genes were detected in any genomes.

### 2.5. Phylogenetic Analysis and Comparison of Antibiotic-Resistance Determinants

**Figure 2:**
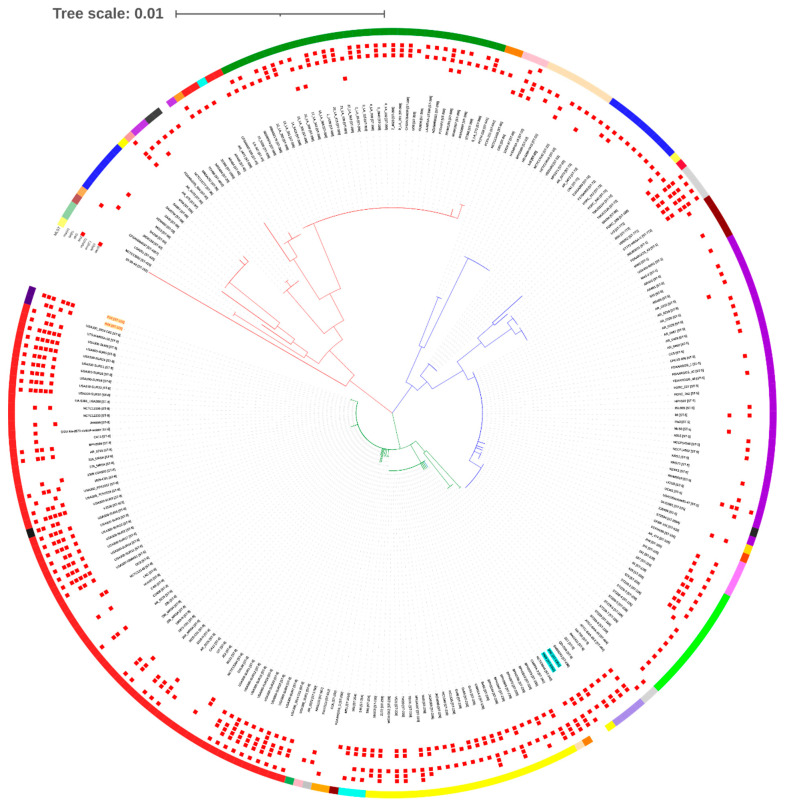
A Maximum Likelihood tree based on variations in housekeeping genes (*arcC*, *aroE*, *glpF*, *gmk*, *pta*, *tpi*, and *yqi*) of the 254 (test strains *n* = 2, global strains *n* = 252) MRSA genomes. The evolutionary history was inferred by using the Maximum Likelihood method and the General Time Reversible model. The tree is drawn to scale, with branch lengths measured in the number of substitutions per site. The tree also shows sequence type (ST) assignment and antibiotic resistance genes. The test strains are highlighted in red, boldfaced, and yellow background. The strains (M92 and V605) for which new ST numbers (ST-5354, and ST-5355) were assigned are boldfaced and highlighted in blue background. The rectangular tree is given in Additional file 1 for better presentation.

**Figure 3:**
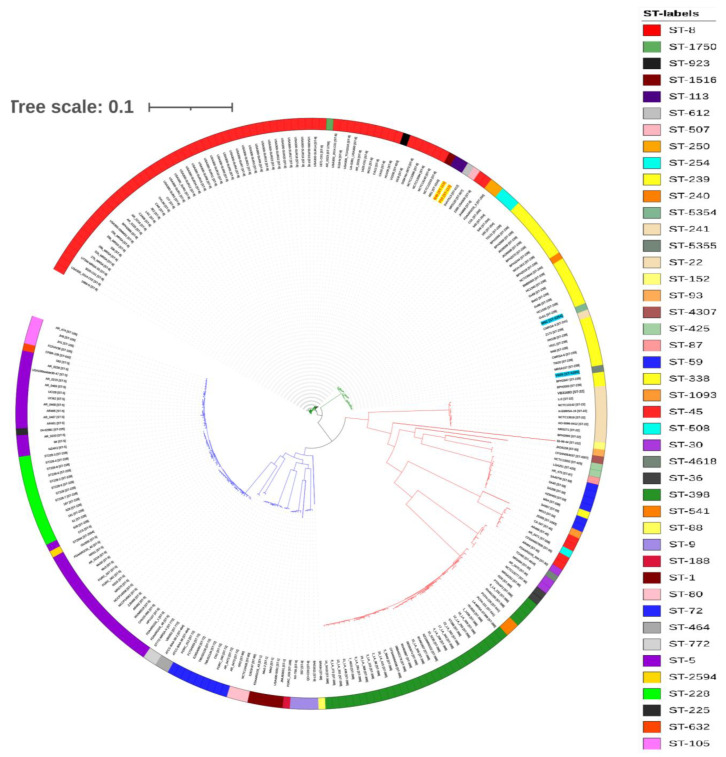
Circularized SNP tree of 253 (test strains *n* = 2, and global strains *n* = 251) MRSA genomes. The Maximum Likelihood tree generated by the single nucleotide polymorphisms (SNPs) with the reference genome USA300_FPR3757, which is not shown in the tree. The test strains are colored in red, boldfaced, and highlighted with a yellow background. Color keys indicating ST numbers are provided alongside the circularized tree.

The MLST- and SNP-based phylogenetic trees (with the reference genome USA300_FPR3757 ST8) divided MRSA strains into three clades (Figure 2 and Figure 3). As expected, the MLST tree clearly clustered each strain in their respective ST clades. The ST113 strains (P10 and R46) were not closely related to any strains and were grouped in a separate clade with ST8 strains at the bottom of the tree (Figure 2). However, the SNP phylogenetic tree grouped ST113 strains (P10 and R46) close to strains M51 ST1516 (CP030137.1), SVH7513 ST612 (CP029166.1), and 2395 USA500 ST8 (CP007499.1) (Figure 3).

The MLST tree annotated with common resistance determinants revealed that ST113 strains (P10 and R46) carry more genes associated with antibiotic resistance compared to their closely related strains M51 ST1516 and SVH7513 ST612. However, they have a similar profile of antibiotic resistance genes to that of ST8 strains (Figure 2). 

### 2.6. Genome-Wide Comparison of Clusters of Orthologous Groups (COGs)

**Figure 4:**
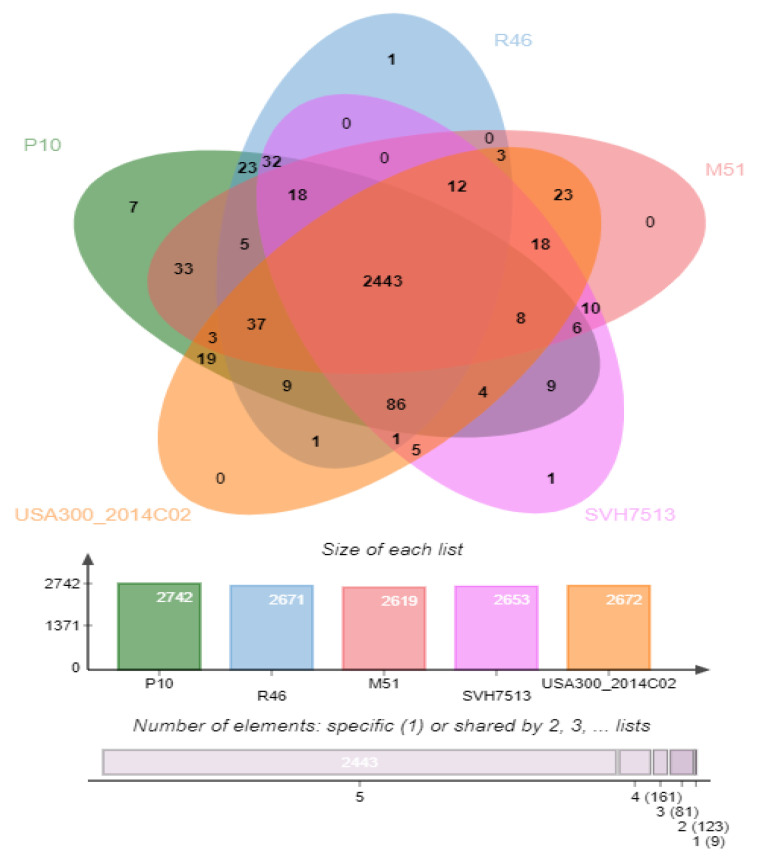
Comparison of clusters of orthologous groups (COGs) of P10 and R46 with their phylogenetically related strains M51 ST1516, SVH7513 ST612, and USA300_2014.C02 ST8. The Venn diagram represents the numbers of unique and shared COGs, while the bar chart represents the number of clusters in each strain.

The orthologous gene analysis identified 2934 proteins and 2742 clusters in P10, 2745 proteins and 2671 clusters in R46, 2638 proteins and 2619 clusters in M51, 2760 proteins and 2653 clusters in SVH7513, and 2778 proteins and 2672 clusters in USA300_2014C02. Among the COGs, 2443 clusters are shared by all the strains (core genome orthologous genes), seven clusters are unique to strain P10, and one cluster is unique to each R46 and SVH7513 strain (Figure 4). Functional enrichment analysis by OrthoVenn2 showed that the COGs unique to strain P10 are involved in ATP binding (GO:0005524), tetracycline resistance (Gene: *tet*, GO:0046677), and two clusters are involved in the transposition of insertion sequence element IS257 in transposon Tn4003 (GO:0032196). The other three unique clusters of strain P10 and one cluster unique to each R46 and SVH7513 did not reveal significant enrichment of any gene ontology terms.

## 3. Discussion

MRSA is an important drug-resistant opportunistic pathogen and a leading cause of a wide variety of infections, resulting in a serious economic burden [22]. MRSA develops resistance against existing antibiotics due to antibiotic selection pressure and/or through horizontal gene transfer (HGT) of mobile genetic elements (MGEs) such as plasmids, prophages, and transposons. It possesses many virulence factors involved in adherence, colonization, and invasion abilities to avoid host immune defense. Furthermore, the emergence of new clones of MRSA in both healthcare settings and the community make the treatment of MRSA infections more challenging [23]. Therefore, genomic epidemiology and pattern of virulence and antibiotic resistance of MRSA clones is required to prevent MRSA infection and for appropriate therapy. Multiple clones of MRSA are circulating in the world, among ST8 (CC8) and ST5 (CC5) are the most ubiquitous and diverse sequence types [24]. However, very limited data are available reporting the molecular epidemiology of MRSA strains from Pakistan. A few studies characterized MRSA isolates based on PFGE, SCC*mec*, and MLST [18,19,20]. However, none of these studies performed whole-genome sequencing of MRSA strains, which makes it difficult to compare MRSA strains in Pakistan to those in other regions of the world. 

Here, we performed whole-genome sequencing of two MRSA strains (P10 and R46) isolated from Pakistan to understand the genetic diversity, sequence type, and distribution of MGEs and genes associated with antibiotic resistance and virulence. The strain P10 was phenotypically resistant to all tested antibiotics and susceptible to chloramphenicol, vancomycin, and rifampicin and strain R46 was susceptible to chloramphenicol, vancomycin, and cefepime. The strains were further confirmed for the presence of *mecA* gene by PCR and were whole-genome sequenced. The sequenced MRSA strains belong to ST113, and this sequence type MRSA strains are also previously reported in Pakistan and India characterized by PFGE, SCC*mec*, and MLST [17,18,20]. The in-silico SCC*mec* typing revealed that strains P10 and R46 ST113 harbor SCC*mec* type IV, and therefore possibly CA-MRSA as SCC*mec* types IV and V are predominantly associated with CA-MRSA [12,13,14]. CA-MRSA are generally susceptible to non-β-lactam antibiotics and possesses the virulence-associated gene Panton-Valentine Leucocidin (PVL), whereas HA-MRSA are associated with nosocomial infections and are generally resistant to non-β-lactam antibiotics and do not possess the PVL gene [25]. The strain P10 contains a plasmid pI5S5 (HE579068.1), an unnamed plasmid “unnamed1” (CP030577.1), and three complete phages of length 42 kbp, 48.4 kbp, and 74.2 kbp. The strain R46 contains a plasmid pRM27 (KT780704.1) and one *S. epidermidis* isolate BPH0662 plasmid (LT614820.1) and two complete phages having genome size 55.6 kb and 71.4 kb (Table 3 and Table 4). The presence of plasmids and phage regions enhances pathogenicity and the capacity of acquiring antibiotic-resistant genes, allowing them to become more virulent and antibiotic-resistant to survive in different environments [26,27].

The strains shared many antibiotic resistance determinants, but strain P10 contains more resistant genes than R46, including two aminoglycoside-resistance genes *aph(3’)-IIIa* and *aad(6)*, and a streptothrin-resistance gene *sat-4* conferring resistance by inactivation of antibiotics, a tetracycline-resistance gene *tet(K)* conferring resistance via the transport of antibiotics out of the cell, a mupirocin-resistance gene *mupA,* and a point mutation in *fusA* conferring resistance to fusidic acid by antibiotic target alteration. On the other hand, a plasmid-associated gene *ant(4’)-Ib* (conferring aminoglycosides resistance by inactivation of antibiotics) is found in strain R46 (Table 1). This potentially reflects that strain P10 is a highly antibiotic-resistant strain, and the genotypic antibiotic resistance profile is highly correlated with the phenotypic resistance. The versatility in virulence factors plays a key role in the pathogenicity of MRSA. For example, MRSA strains containing *pvl*, *hla*, *tsst*, and *fnbA,* cause skin and soft tissue infection, sepsis, and necrotizing pneumonia [2]. The identification of virulence factors revealed that strains P10 and R46 share many common virulence-associated genes, and strain R46 has an additional Ser-Asp rich fibrinogen-binding protein (*sdrE*) (Figure 1). However, no Panton-Valentine leucocidin (*lukF-PV/lukS-PV*) and toxic shock syndrome toxin (*tsst*) genes were detected in any genomes. The *S. aureus* Panton-Valentine leucocidin (PVL), encoded by *lukF-PV* and *lukS-PV*, is a pore-forming toxin that has been strongly associated with necrotizing pneumonia and skin infections [28]. The presence of Panton-Valentine leucocidin (*lukF-PV/lukS-PV*) gene in combination with toxic shock syndrome toxin (*tsst*) causes toxic shock syndrome toxin-1 (TSST-1) [29], but the absence of both virulence genes in ST113 strains (P10 and R46) may indicate that these strains are less pathogenic. Previous studies also reported PVL negative SCC*mec* type IV MRSA strains from this region [30,31,32]. Fibronectin binding proteins (*fnbA* and *fnbB*) provide strong adherence properties to *S. aureus* [33]. However, P10 and R46 strains harbor *fnbA* while *fnbB* is absent in both the strains. 

Based on the holistic comparison of phylogenetic trees, the SNP phylogeny was found to be concordant to MLST-based phylogeny and provided comparable results. The MLST phylogenetic tree very clearly grouped each strain in their respective ST clades. Since other ST113 strains are not available for comparison, hence ST113 strains P10 and R46 were grouped in a separate clade with ST8 strains at the bottom of the tree (Figure 2). However, SNP phylogenetic tree grouped ST113 strains P10 and R46 close to strains M51 ST1516 (CP030137.1), SVH7513 ST612 (CP029166.1), and 2395 USA500 ST8 (CP007499.1) (Figure 3). This suggests that ST113 strains are closely related to ST8 strains, and on close observation we found that ST113 is a single-locus variant (*tpi* locus) of ST8. In addition, ST8 strains also harbor SCC*mec* types IV encoding *mecA* gene [34]. However, ST113 strains (P10 and R46) are PVL negative. The MLST tree annotated with common resistance determinants revealed that ST113 strains (P10 and R46) carry more antibiotic resistance genes compared to their closely related strains M51 ST1516 and SVH7513 ST612. However, they have a similar profile of antibiotic resistance genes to that of ST8 strains (Figure 2). The orthologous genes analysis identified 2443 clusters shared by ST113 and closely related strains. The strain P10 has seven unique clusters involved in ATP binding, tetracycline resistance (tetracycline-resistant gene *tet*), and a transposase for the insertion of sequence element IS257 in transposon Tn4003. This possibly makes P10 more resistant to antibiotics and may help in acquiring more genes via HGT.

## 4. Conclusions

The MRSA strains P10 and R46 isolated from Pakistan belong to ST113 and harbor SCC*mec* type IV. The antibiotic resistance determinants in strain P10, excluding those shared with strain R46, include two aminoglycoside-resistance genes *aph(3’)-IIIa* and *aad(6)*, a streptothrin-resistance gene *sat-4*, a tetracycline-resistance gene *tet(K)*, a mupirocin-resistance gene *mupA*, and a point mutation in *fusA* conferring resistance to fusidic acid and strain R46 has an additional plasmid associated gene *ant(4’)-Ib*. The phylogenetic relationship of P10 and R46 with other MRSA strains suggests that ST113 strains are closely related to ST8 strains and ST113 strains are a single-locus variant of ST8. However, ST113 strains are PVL negative. These findings provide important information of MRSA clone ST113 in Pakistan, and these strains can be used as reference strains for the comparative genomic analysis of other MRSA strains in Pakistan and ST113 strains globally. 

## 5. Methods

### 5.1. Bacterial Isolates Collection and Antibiotic Susceptibility Testing

The isolate P10 was collected from Khyber Teaching Hospital, Peshawar and R46 was collected from Railway General Hospital, Rawalpindi/Islamabad in January–April 2019. The P10 was isolated from a pus sample and R46 from a urine sample, and both were cultured on mannitol salt agar (MSA) plates and incubated at 37 °C for 24 h. The preliminary identification of the isolates was performed using biochemical (catalase, oxidase, and coagulase tests) characteristics [35]. The antimicrobial susceptibility testing was performed by the disc diffusion method as per CLSI 2015 guidelines for the following antibiotic classes: β-lactams (ampicillin (10 µg), methicillin (10 µg), and oxacillin (1 µg)), aminoglycosides (gentamicin (10 µg) and streptomycin (25 µg)), macrolide (erythromycin (15 µg)), lincomycin (clindamycin (2 µg)], oxazolidinones [linezolid (30 µg)), cephalosporins (cefixime (5 µg), and cefepime (30 µg)), carbapenems [meropenem (10 µg)), chloramphenicols (chloramphenicol (30 µg)), tetracyclines (tetracycline (30 µg)), fluoroquinolones (ciprofloxacin (5 µg)), glycopeptides (vancomycin (5 µg)), rifampicins (rifampicin (5 µg)), and fusidane (fusidic acid (10 µg)). The *mecA* gene was amplified using primers *mecA*-F 5′-AAAATCGATGGTAAAGGTTGGC-3′ and *mecA*-R 5′-AGTTCTGGAGTACCGGATTTGC-3′) to confirm the nature of methicillin resistance [36]. 

### 5.2. Whole-Genome Sequence Analysis and Molecular Typing

Genomic DNA was extracted from overnight broth cultures by Invitrogen^®^ DNA extraction kit as per the manufacturer’s instructions. The extracted genomic DNAs were quantified by Qubit 2.0 fluorometer, and integrity was checked by 0.75% agarose gel electrophoresis. The whole-genome sequencing of the strains was performed on the Illumina Hiseq 2500 platform by MicrobesNG, Birmingham. The sequencing raw reads were trimmed using Trimmomatic, and the resultant reads were assembled into contigs using SPAdes [37]. The generated contigs were used to confirm the *S. aureus* isolates using the SpecieFinder 2.0 at Center for Genomic Epidemiology (CGE) available at https://cge.cbs.dtu.dk/services/SpeciesFinder/, accessed on 12 February 2021. The assembled genomes were annotated with NCBI prokaryotic genome annotation pipeline [38]. The contigs were aligned and reordered with *S. aureus* reference genome NCTC8325 using Mauve and were subjected for further analysis [39]. The in silico multi-locus sequence typing (MLST), *spa* typing, and SCC*mec* element were determined by using MLST 1.6 (https://cge.cbs.dtu.dk/services/MLST/, accessed on 12 February 2021), SCC*mec*Finder 1.2 (https://cge.cbs.dtu.dk/services/SCCmecFinder/), accessed on 12 February 2021, and *SpaTyper* 1.0 (https://cge.cbs.dtu.dk/services/spatyper/, accessed on 12 February 2021), respectively. The genomes were visualized using CGView server (http://cgview.ca/, accessed on 28 June 2021) [40].

### 5.3. Identification of Plasmids, Prophages, and Genes Associated with Antibiotic Resistance and Virulence

Plasmid replicons and prophages in the sequenced genomes were identified by plasmidFinder and PHASTER, respectively [41,42]. The ResFinder at CGE (https://cge.cbs.dtu.dk/services/ResFinder/, accessed on 16 February 2021) [43] and the comprehensive antibiotic resistance database (CARD; https://card.mcmaster.ca/, accessed on 16 February 2021) [44] were used to identify antibiotic resistance determinants. The virulence-associated genes were identified and annotated based on the Virulence Factor Database (VFDB) at http://www.mgc.ac.cn/VFs/, accessed on 16 February 2021 [45].

### 5.4. MLST and SNP Based Phylogenetic Analysis

MRSA strains (*n* = 252) available from the NCBI database were used to draw MLST- and whole-genome SNP-based phylogenetic trees to understand the genetic diversity of the sequenced ST113 MRSA strains. The MLST analysis of all the strains was conducted with the following scheme which comprises seven housekeeping genes: *arcC*, *aroE*, *glpF*, *gmk*, *pta*, *tpi,* and *yqi*. The full-length gene sequences were extracted from each genome using MLST version 1.6 integrated into batch upload service provided by the Center for Genomic Epidemiology [46]. The allelic profiles were determined for each strain and new STs were assigned to the strains with unknown STs. Multiple sequence alignment was performed on the concatenated sequences of housekeeping genes using MUSCLE [47], and a maximum likelihood tree with 1000 bootstrap iterations was constructed using MEGA X [48]. 

For SNP-based phylogenetic analysis, the genomes were submitted to CSI phylogeny 1.4 online server at https://cge.cbs.dtu.dk/services/CSIPhylogeny/, accessed on 25 February 2021 [49] with the following default setting: minimum depth at SNP positions 10, relative depth at SNP positions 10, the minimum distance between SNPs (prune) 10, minimum SNP quality 30, and minimum Z-score of 1.96. The SNPs were called against MRSA strain USA300_FPR3757, concatenated, and multiple-aligned. A maximum-likelihood tree was generated based on concatenated SNP alignment using the FastTree 2 tool [50].

### 5.5. Comparative Analysis of Whole-Genome Orthologous Clusters

The genome-wide comparison and annotation of clusters of orthologous groups (COGs) of P10 and R46 strains with their phylogenetically related strains (M51 ST1516; CP030137.1, SVH7513 ST612; CP029166.1, and USA300_2014.C02 ST8; CP012120.2) were performed using OrthoVenn2 software [51]. An E-value (cut-off) of 1 × 10^−5^ for protein similarity comparisons and an inflation value of 1.5 for the generation of orthologous clusters were used.

## Figures and Tables

**Table 1:** Phenotypic and genotypic antibiotic resistance profile of MRSA strains P10 and R46.

Antibiotic	P10		R46	
	PR	GR	PR	GR
Ampicillin	R	*blaZ*, *mecA*, *mecR1*	R	*blaZ*, *mecA*, *mecR1*
Methicillin	R		R	
Oxacillin	R		R	
Gentamicin	R	*aac(6′)-Ie-aph(2″)-Ia*, *aph(3’)-IIIa,aad(6)*	R	*aac(6′)-Ie-aph(2″)-Ia*, *ant*(*4’*)*-Ib*
Streptomycin	R		R	
Erythromycin	R	*erm(C)*	R	*erm*(*C*)
Clindamycin	R		R	
Linezolid	R		R	
Cefixime	R	ND	R	ND
Cefepime	R	ND	S	ND
Meropenem	R	ND	R	ND
Chloramphenicol	S	ND	S	ND
Tetracycline	R	*mepA*, *tet(38)*, *tet(K)*, *mepR*	R	*mepA*, *tet(38)*, *mepR*
Ciprofloxacin	R	*gyrA*, *parC*	R	*gyrA*, *parC*
Rifampicin	S	ND	R	ND
Fusidic acid	R	*fusA*	R	ND
Vancomycin	S	ND	S	ND
Trimethoprim	ND	*dfrG*	ND	*dfrG*
Streptothrin	ND	*sat-4*	ND	ND
Mupirocin	ND	*mupA*	ND	ND
Fosfomycin	ND	*murA*	ND	*murA*

(PR = Phenotypic Resistance, GR = Genotypic Resistance, R = Resistant, S = susceptible, ND = not determined).

**Table 2:** Genomic features and characteristics of MRSA strains P10 and R46.

Characteristics	P10	R46
Genome size (bp)	2,955,291	2,822,631
Contigs	90	72
GC content %	32.7	32.7
N_50_	84,395	84,399
N_75_	52,815	50,022
L_50_	10	11
Largest Contig (bp)	294,580	261,096
N50	84,395	84,399
No. of CDS	3031	2827
No. of tRNA	54	55
No. of rRNA	9	10
ST	113	113
SCC*mec* type	IV	IV
*spa*-type	t064	unknown
NCBI Accession number	JAHHEA000000000.1	JAHKSM000000000.1

**Table 3:** Summary of plasmids present in MRSA ST113 strains P10 and R46.

Strain	Plasmid	Total Length	Most Similar Plasmid	% Similarity	Accession Number
P10	1	2375 bp	pI5S5	99.78	HE579068.1
	2	2855 bp	unnamed1	99.72	CP030577.1
R46	1	2494 bp	*Staphylococcus epidermidis* isolate BPH0662 plasmid: 1	99.83	LT614820.1
	2	2250 bp	pRM27	100	KT780704.1

**Table 4:** Characteristics of complete prophages present in MRSA ST113 strains P10 and R46.

Strain	Region	Region Length	Total Proteins	Phage Hit Proteins	GC %	Specific Keywords	Most Common Phage
P10	1	48.4 kbp	64	64	35.02	recombinase, terminase, portal, head, capsid, tail	Staphy_SA13_NC_021863
	2	74.2 kbp	79	69	32.84	tail, capsid, head, portal, integrase	Staphy_phi2958PVL_NC_011344
	3	42 kbp	46	29	32.66	integrase, portal, protease, capsid, head, tail	Staphy_phiN315_NC_004740
R46	1	55.6 kb	69	67	32.79	integrase, terminase, portal, protease, head, tail	Staphy_phiNM3_NC_008617
	2	71.4 kb	76	68	32.77	tail, capsid, head, portal, integrase	Staphy_phi2958PVL_NC_011344

## Data Availability

The whole-genome sequence and raw reads data of the strains reported in this study have been deposited in NCBI GenBank and SRA, respectively, under the Bioproject accession number PRJNA520768.

## Supplementary Materials

The following are available online at https://www.mdpi.com/article/10.3390/antibiotics10091121/s1, Additional file 1: A rectangular MLST based phylogenetic tree annotated with sequence type (ST) and genotypic antibiotic resistance profile of MRSA strains.

## Abbreviations

CARD: Comprehensive Antibiotic Resistance Database, HGT: Horizontal Gene Transfer, MLST: Multi-locus Sequence Typing, MRSA: Methicillin-resistant *Staphylococcus aureus*, SCC*mec*: Staphylococcal Cassette Chromosome *mec*, PFGE: Pulsed-field Gel Electrophoresis, SNP: Single Nucleotide Polymorphism, VFDB: Virulence Factor Database, WGS: Whole-genome Sequence.
