# Supplementary figures and images for: Genomic Investigation of Methicillin-Resistant Staphylococcus aureus ST113 Strains Isolated from Tertiary Care Hospitals in Pakistan
Source: Antibiotics (Basel). 2021 Sep 17;10(9):1121. doi: 10.3390/antibiotics10091121 (PMC8465543; doi:10.3390/antibiotics10091121)

Tree scale: 0.001

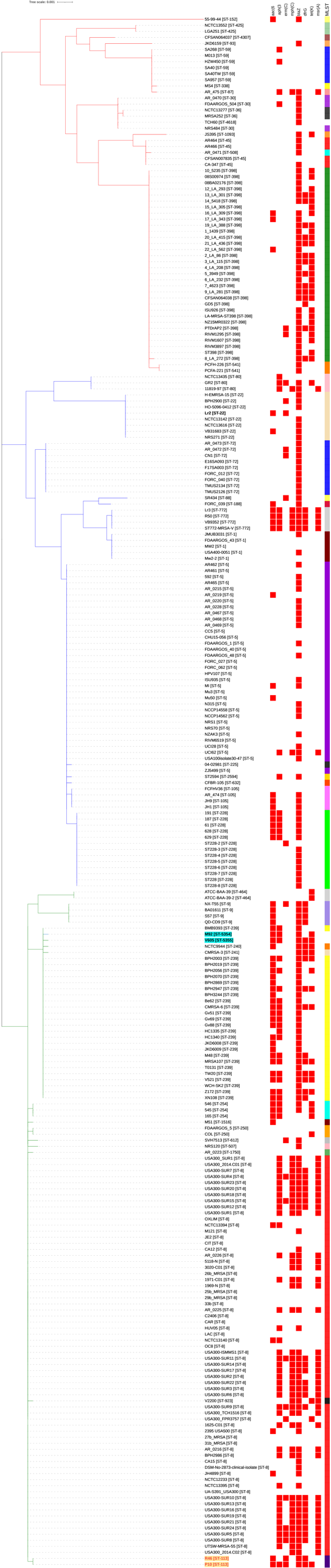

Supplement: Supplementary file 1 [file antibiotics-10-01121-s001.zip › antibiotics-1355907-supplementary.pdf]
